# Regulation of Peptaibol Profile by Velvet LAE1/VEL1 in *Trichoderma* Species During In Vitro Confrontations with *Fusarium graminearum*

**DOI:** 10.3390/microorganisms14040847

**Published:** 2026-04-09

**Authors:** Yaqian Li, Hui Zhang, Huimin Ji, Wanping Zhou, Xinhua Wang, Jie Chen

**Affiliations:** 1 School of Agriculture and Biology, Shanghai Jiao Tong University, Shanghai 200240, China; yaqianli2008@163.com (Y.L.); zhanghui0121@sjtu.edu.cn (H.Z.); 17730197719@163.com (H.J.); xhwang@sjtu.edu.cn (X.W.); 2 State Key Laboratory of Microbial Metabolism, Shanghai Jiao Tong University, Shanghai 200240, China; wpzhou@sjtu.edu.cn

**Keywords:** *Trichoderma*, *Fusarium graminearum*, peptaibols, velvet LAE1/VEL1, in vitro confrontation

## Abstract

Peptaibols, predominantly secreted by *Trichoderma* species, are a class of linear peptides composed of five to twenty amino acid residues, synthesized non-ribosomally and enriched with α-amino isobutyric acid. These unique peptides appear to be highly effective in mediating the interactions between *Trichoderma* and plant pathogenic fungi. In this study, Ultra-Performance Liquid Chromatography–Quadrupole Time-Of-Flight Mass Spectrometry/Mass Spectrometry (UPLC-QTOF-MS/MS) technology was used to detect peptaibols profiles of *Trichoderma* strains during their interactions with the pathogen *Fusarium graminearum*. MS investigations of crude extracts derived from in vitro confrontations of *Trichoderma atroviride* T23 and its genetically modified counterparts, dual-culture assays of Mlae1, Mvel1, OElae1, and OEvel1 with *F. graminearum* were performed to shed light on the regulatory role of the velvet complex composed of LAE1&VEL1 in the synthesis of peptaibols during the microbial interaction. These results revealed intriguing variations in the total peptaibols produced during the interactions, as well as some differences in the specific peptaibol profiles between the confrontation and control tests. The overexpression strains, OElae1 and OEvel1, distinguished themselves by their proficiency in inducing long-residue peptaibols synthesis, attaining an impressive biocontrol index of up to 76%. The crude extracts containing peptaibols of OElae1 and OEvel1 demonstrated a capability to enhance cell membrane permeability and decrease DON toxin production in *F. graminearum*, and the crude extracts of OElae1 strains exhibited more effectiveness in reducing DON toxin production. In conclusion, the interaction with *F. graminearum* significantly impacted the peptaibol production in the examined *Trichoderma* strain, emphasizing the intricate interplay and reciprocal influence of genetic factors and environmental stimuli.

## 1. Introduction

*Fusarium graminearum*, a prevalent filamentous fungus, causes devastating crop diseases, notably stalk rot in corn and Fusarium Head Blight (FHB) in wheat, and is a global threat to food security. *F. graminearum* affects more than 4.5 million hectares of wheat annually on average, accounting for about 20% of the total wheat planting area, and caused a yield loss of more than 3.41 million tons per year from 2000 to 2018 in China [1,2]. Between the early 1990s and 2008, FHB caused an estimated $3 billion in yield losses. By

Producing toxins like deoxynivalenol (DON), it inhibits plant growth, causing head blight in wheat that can result in crop loss, and leads to root and head rot in other cereals like corn, barley, and oats. The incidence rates of corn stalk rot vary greatly in different years and regions. In normal years, the incidence rate ranges from 10 to 20%. However, under appropriate conditions in years favorable for the disease, the incidence rate can reach over 50%, and in some cases, it may even lead to complete crop loss [3,4]. Chemical control offers rapid and stable effects, yet it risks inducing resistance and causing environmental pollution. In contrast, biological control is an alternative to chemical control for the mitigation of plant diseases.

Biological control is an eco-friendly approach that uses living organisms or their metabolites to control diseases and pests. *Trichoderma* fungi are effectively marketed worldwide as biocontrol agents (BCA) on numerous crops due to the secretion of lytic cell wall-degrading enzymes (CWDEs) and the production of a broad spectrum of secondary metabolites against *F. graminearum* [5,6,7]. Many *Trichoderma* species have been isolated, and their biocontrol role in the biological control of plant disease was reviewed by Yao et al. [8]. For example, *T. asperellum* GDFS1009 has been reported to significantly inhibit *F. graminearum* and effectively control maize stalk rot under both greenhouse and field conditions [9]. Tian et al. (2018) demonstrated the capacity of *T. asperellum*, *T. atroviride*, and *T. harzianum* to inhibit the growth of *F. graminearum* mycelia in vitro, and reduced the biosynthesis of Zearalenone (ZEN) and zearalanone (ZAN) [10]. More recently, a report by Pedrero-Méndez et al. revealed that *T. asperellum* T25 and *T. harzianum* T136 can inhibit *F. graminearum* growth and activate salicylic acid-dependent defense pathways in wheat [11]. By clinging to, twining around, and penetrating the hyphae of *F. graminearum*, *Trichoderma* mycelium effectively outcompeted its counterpart, causing progressive deformation and disappearance of the latter [12].

It is well known that *Trichoderma* species are prolific producers of diverse secondary metabolites against phytopathogens, such as peptaibols, polyketides, and terpenes, exhibiting broad-spectrum antimicrobial properties and unique potential for agricultural applications [7,13,14]. Peptaibols are typically composed of 5 to 21 amino acid residues. They exhibit diverse biological activities such as antifungal activity, which arise from their specific amino acid sequences and three-dimensional structures. Peptaibols are linear and amphipathic peptides synthesized by multi-enzyme system non-ribosomal peptide synthetases (NRPSs), which are composed of multiple modules arranged in a specific spatial order [15]. Genome analysis revealed that there are up to three types of NRPSs with 7, 14, or 18–20 amino acid incorporation modules in *Trichoderma*. Alamethicin (ALM) is the first peptaibol reported from *Trichoderma viride* and has been the most extensively studied, owing to its pronounced antimicrobial activity and ability to induce plant resistance [16]. The primary mode of action for peptaibols involves the formation of ion channels, which disrupts membrane integrity, causes cytoplasmic leakage, and induces cell death [17,18]. *Trichoderma* spp. are a dominant genus that can produce structural diversity and bioactivity of peptaibol compounds that inhibit a variety of plant pathogenic fungi and can also cooperate with cell wall-degrading enzymes on pathogenic fungi to effectively inhibit pathogen growth [19]. Supporting this, Zhao et al. and Song et al. reported that specific peptaibol trichokonins are effective against a broad range of plant pathogens, including *F. oxysporum* [20,21]. Novel 9-residue peptaibols, koningiopsins from *T. koningiopsis,* share sequence similarity with trikoningins and related peptaibols, showing differential antimicrobial activities against bacteria, yeasts, and fungi, likely due to differences in cell wall architecture [22]. In recent years, despite strong interest in the identification of peptaibols and screening for new components, there exist numerous gaps in our basic understanding of how they are produced and how they work in practical applications.

The regulation of peptaibol biosynthesis pathways is complex and involves several interconnected networks. The velvet family of regulatory proteins plays a pivotal role in coordinating fungal secondary metabolism and developmental processes, including both asexual and sexual sporulation. LaeA, a putative S-adenosyl methionine (SAM)-dependent methyltransferase, was initially identified in *Aspergillus* as a global regulator of secondary metabolism [23,24,25]. In *Trichoderma* species, the LaeA homolog LAE1 has emerged as a master regulator of cellulolytic enzyme production and secondary metabolism [26]. Karimi et al. further revealed that LAE1 positively regulates 17 polyketide synthases and seven non-ribosomal peptide synthetases in *Trichoderma reesei*, including those responsible for peptaibol biosynthesis [27]. Supporting these findings, Shi et al. demonstrated that TLlae1 in *Trichoderma longibrachiatum* SMF2 regulates sporulation efficiency and secondary metabolite production, with knockout strains showing significantly reduced trichokonin biosynthesis through the downregulation of TLX1 and TLX2 peptaibol synthetase genes [28]. The VeA component of the Velvet complex similarly influences fungal morphogenesis and secondary metabolism [29]. Mukherjee et al. first characterized the *veA* homolog *vel1* in *Trichoderma virens*, demonstrating its role in conidiation, chlamydospore formation, and suppression of secondary metabolite gene clusters, including NRPSs, PKSs, and methyltransferases [30]. Interestingly, expression of the NRPS synthetase gene for paracelsin biosynthesis is elevated in both *lae1* knockout and *lae1*-overexpressing strains of *T. reesei* [27]. Our previous research showed that *Lae1* in *T. atroviride* T23 affects primary metabolites, phospholipid, as well as the regulation of secondary metabolites in *T. atroviride* [31]. Similarly, *vel1* governs sporulation, secondary metabolism, mycoparasitism, and biocontrol against *Fusarium graminearum* [32]. Overexpression of the *vel1* gene in *T. asperellum* enhanced cellulase and xylanase activities and resistance against *F. verticillioides* [33]. While the velvet complex is a known global regulator of secondary metabolite biosynthesis, the specific regulation of peptaibols production in response to pathogen presence is yet to be elucidated.

In this study, we investigate the functional crosstalk between velvet-mediated regulation and antifungal activity by analyzing peptaibol production dynamics during interactions between *T. atroviride* 23 (and its Mlae1, Mvel1, OElae1, and OEvel1 deletion and overexpression mutants) with *F. graminearum*. By correlating genetic modifications in velvet components with peptaibol profiles and antagonistic efficacy, we aim to elucidate the role of peptaibols in fungal competition and establish the regulatory hierarchy of velvet proteins in biocontrol processes. This work provides insights for developing novel plant protection strategies while advancing our understanding of molecular dialogs between *Trichoderma* and *Fusarium.*

## 2. Materials and Methods

### 2.1. Fungal Strains and Culture Conditions

The wild-type of *Trichoderma atroviride* T23, *lae1* deletion mutant (Mlae1), *vel1* deletion mutants (Mvel1), *lae1* overexpression mutants (OElae1), T23 *vel1* overexpression mutants (OEvel1), and *F. graminearum* were obtained from our previous study [31,32]. All strains were cultivated on either Potato Dextrose Agar (PDA) or on Malt Extract Agar (MEA). All cultures were stored at −80 °C in glycerol stocks.

### 2.2. Direct Confrontation Assays

In vitro antagonistic properties of the five *Trichoderma* strains were investigated based on the method described by Ji et al. and Szekeres et al. [34,35]. Briefly, the experiments were performed in three parallel inoculations for *Trichoderma*–plant pathogenic fungus combinations. Plates containing *F. graminearum* or the *Trichoderma* strains alone were used as controls. Single agar plugs from the freshly growing mycelium of were inoculated onto the surface of Petri plates (15 cm in diameter) containing MEA, at a position 4.5 cm from the center of the plate. The plates were kept at a 28 °C incubator in a 16/8 light/darkness. After 7 days, the visible area of the *Trichoderma* colony and the total area occupied by both the colonies of *Trichoderma* and *F. graminearum* were measured. The growth areas were quantified from digital images (TIFF format) using ImageJ 1.x. Images were converted to 8-bit grayscale and background corrected. The scale was set using a reference bar. Colony area was determined via threshold segmentation (Otsu’s method). In confrontation assays, the pathogen area was selectively measured using the polygon selection tool. The antifungal inhibition rate (%) was calculated as [(Ac − At)/Ac] × 100, where Ac and At are the average pathogen areas in control and dual-culture plates, respectively. Three biological replicates were analyzed per treatment.

### 2.3. Dry Weight of Mycelium

*Trichoderma* and *F. graminearum* were cultured in MEA using the dual-culture method. One 5 mm mycelial plug was taken from the growing margin and inoculated into 250 mL Erlenmeyer flasks containing 100 mL of sterile PDB. All treatments were incubated at 28 ± 1 °C on an orbital shaker at 180 rpm for 7 days in darkness. The mycelia were filtered under vacuum through a pre-dried and pre-weighed (W1) Whatman No. 1 filter paper; the mycelial mat on the filter was thoroughly rinsed with 200 mL of sterile distilled water. The filter paper with the harvested mycelium was carefully transferred to a labeled, pre-weighed glass Petri dish. The samples were placed in a forced-air drying oven at 70 °C for 48–72 h until a constant weight was achieved. The final dry weight (W2) was measured immediately after cooling using an analytical balance. The mycelial dry weight for each sample was calculated as W2 − W1. Mycelial dry weight, measured from 100 mL aliquots, was normalized to grams per liter.

### 2.4. Peptaibol Extraction

The mycelium and conidia were scraped from the agar surface and ground three times with liquid nitrogen. A total of 5 mL of chloroform was added to steep and then extract peptaibols twice. Transfer the solution to a 1.5 mL centrifuge tube and concentrate under vacuum at 45 °C until complete evaporation. Add 187 μL of methanol to each 1.5 mL centrifuge tube, centrifuge at 12,000 rpm for 2 min, and transfer the supernatant to a new 1.5 mL centrifuge tube. Concentrate under vacuum until the liquid is completely evaporated, then add 200 μL of methanol. Store the extract at −20 °C for use.

### 2.5. Analytical Procedures Peptaibols Using Acquity UPLC-QTOF-MS

UPLC-QTOF-MS was performed using a Waters ACQUITY UPLC system equipped with a Micromass Q-TOF Premier mass spectrometer (Waters MS Technologies, Manchester, UK). Chromatographic separations were performed on a 2.1 × 100 mm (1.7 μm) ACQUITY BEH C18 chromatography column. The column temperature was set at 45 °C, and the gradient eluting program was started from 5% B, changed to 20% B within 2 min, to 100% B within 10 min, then changed to 100% B in another 2min, to 95% B in 15 min and, at last, held at 95% B for 4 min (Solvent A: aqueous solution of 0.1% formic acid; Solvent B: Acetonitrile (ACN) of 0.1% formic acid). The total flow rate was 0.40 mL/min. Mass analysis was performed using a Q-TOF mass spectrometer equipped with an ESI source operating in the positive and negative ion modes. The desolvation and cone gas rates were set at 900 L/h at a temperature of 350 °C and 50 L/h, respectively. The source temperature was set at 115 °C. The collision energy for the MS scan was 6 eV; for the MS/MS scan, the collision energy ramped up from 20 eV to 30 eV. Data were acquired in the centroid mode from the mass-to-charge ratio (*m*/*z*) 50 to 2000 at a scan time of 0.5 s with a lock spray frequency of 15 s, and the acquisition mode was MSE.

The signal intensity of each peptide ion was detected by the mass spectrometer. Total peptaibol production was quantified from the total peak area in LC-MS data. The MS/MS response values, represented by the chromatographic peak areas, were analyzed to determine the relative abundance of the peptides. As the response value is proportional to concentration but not an absolute measure, relative quantification was performed based on a comparison of the chromatographic peak area obtained with the characteristic ions of each peptaibol [36,37].

### 2.6. Identification of Peptaibols and Data Analysis

Characteristic *m*/*z* values of the protonated [M + H]^+^ and doubly charged [M + 2H]^2+^, [M + NH_4_]^+^, [M-H_2_O + H] ^+^, and [M + Na]^+^ pseudomolecular ions in the mass spectra were generally observed, which confirmed the molecular mass of each detected compound. Furthermore, MS2 investigations were needed. Arising from the cleavage of the Aib-Pro bond, peptaibols can be divided into a series of fragment ions (b1–b12) related to the N-terminal parts of the peptaibol sequences and a C-terminal fragment ion (y6/y7). Therefore, the first 12 N-terminal residues of the detected peptaibols and the acylium ions (y6/y7) were identified. The novelty of the determined sequences was validated by using “The Comprehensive Peptaibiotics DB”, a Peptaibols Database. (https://peptaibiotics-database.boku.ac.at/downloads/TheComprehensivePeptaibioticsDB_1.0.0.zip 12 August 2022) [38]. As no amino acid analysis was carried out for the determination of the Val/Iva and Leu/Ile isomers, the Vxx/Lxx nomenclature was used in the peptaibol sequences.

### 2.7. Cell Membrane Permeability Test

The fungal preparation and membrane permeability assessment were conducted using a modified protocol based on the study by [39]. *Fusarium graminearum* was initially cultured on PDA at 28 °C for 5 days. Mycelial plugs (5 mm diameter), collected from colony peripheries, were inoculated into 250 mL Erlenmeyer flasks containing 100 mL of potato dextrose broth at a density of four plugs per flask. Cultures were incubated under orbital shaking (200 rpm) at 25 °C for 72 h until reaching late-log phase growth. Mycelia were sequentially rinsed with sterile PBS (pH = 7.4) and surface moisture was removed with Whatman^®^ No.1 filter paper. Precise weighing of fresh mycelial mass: Weigh 1.0 g of mycelium into a 50 mL centrifuge tube, add 30 mL of deionized water and 60 μL of the test crude extract, with 0.2% methanol used as the control. Membrane damage was assessed by monitoring electrolyte leakage, which was measured via an electrical conductivity meter (DDS-307A, INESA Scientific Instrument Co., Ltd., Shanghai, China) at 0, 4, 8, 18, 24, and 48 h. Relative electrolyte leakage (%) was calculated as the equation, where *C*_0_ = initial conductivity, *C_t_* = timepoint conductivity, and *C_total_* = conductivity.

(1) $$Leakage=\left(\frac{C_{t}-C_{0}}{C_{total}-C_{0}}\right)\times 100$$

### 2.8. Detection of Deoxynivalenol (DON)

Scraping 0.1 g of *Fusarium graminearum* treated with peptaibols extraction was homogenized in 500 μL of extraction solvent (10% methanol/water, *v*/*v*) using a Precellys^®^ 24 tissue homogenizer (Bertin Technologies, Montigny-le-Bretonneux, France) (3 × 30 s cycles at 6500 rpm, 4 °C). After 10 min of vortex mixing (200 rpm) and subsequent centrifugation (12,000× *g*, 15 min, 4 °C), supernatants were filtered through 0.22 μm nylon membranes (Millipore^®^, Burlington, MA, USA) prior to analysis. DON standard (Sigma-Aldrich, St. Louis, MO, USA, purity ≥ 98%) stock solution (1 mg/mL in acetonitrile) was serially diluted with extraction solvent to create calibration curves at 20, 40, 80, and 100 μg/mL. Working standards were stored at −80 °C in amber vials to prevent photodegradation. The detection method for vomitoxin refers to the method by Wang et al. [40]. Chromatographic separation was performed on a Waters ACQUITY UPLC^®^ BEH C18 column (Milford, MA, USA) (2.1 × 100 mm, 1.7 μm) maintained at 40 °C. Mobile phases consisted of A: 0.1% formic acid in water; B: 0.1% formic acid in acetonitrile. The gradient elution program was 0–2 min: 5% B → 2–8 min: 5–95% B 8–10 min: 95% B flow rate: 0.3 mL/min; injection volume: 5 μL. DON concentrations were normalized to fungal biomass using:

(2) $$C_{normalized}=\frac{C_{measured}}{W_{mycelia}}\times V_{extract}$$

### 2.9. Statistical Analysis

In order to determine the significant differences between the peptaibol production of *Trichoderma* isolates in the confrontation assays with *F. graminearum* in comparison to the respective controls, peptaibol percentage level (the graph area of a specific peptaibol compound divided by the area of the total peptaibol production) changes were calculated by each peptaibol peak area with the MS Data Review software Mass Frontier 8.0. All data were analyzed for statistical significance by Graphpad Prism software version 10.1.2 (GraphPad Software, www.graphpad.com). Statistical analyses were performed using GraphPad Prism 9. Multiple group comparisons were analyzed by one-way or two-way ANOVA followed by Tukey’s post hoc test. Data are presented as mean ± SD, with significance levels denoted as * *p* < 0.05, ** *p* < 0.01.

## 3. Results

### 3.1. Overexpression of lae1 Enhances Antagonism Against F. graminearum

**Figure 1:**
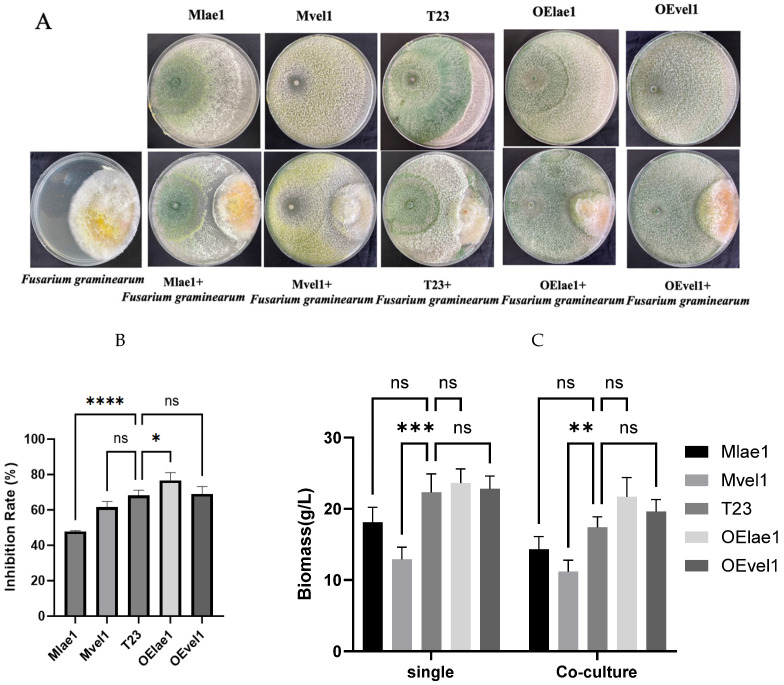
*Trichoderma* species (Mlae1, Mvel1, T23, OEvel1, and OElae1) exhibited distinct growth phenotype zones when co-cultured with *F. graminearum* in the confrontation assays on PDA plates: (**A**) the growth inhibition zone; (**B**) the inhibitory rate; (**C**) the biomass accumulation for the single culture and interaction culture. Significant differences (**** *p* < 0.0001, *** *p* < 0.001, ** *p* < 0.01, * *p* < 0.05, ns, *p* > 0.5).

In the dual-culture assay, *Trichoderma* T23, OElae1, and OEvel1 significantly overgrew on *F. graminearum,* inhibiting its mycelial growth and reducing colony diameter extension; in contrast, Mlae1 and Mvel1 showed less overgrowth and were weaker against *F. graminearum*. As shown in Figure 1B, the OElae1strain had a greater inhibition rate than the other strains, reaching 76.5%, followed by T23 and the OEvel1 strain, which reached 68.1% and 69.0%. These results also indicated that overexpression of *lae1* increased *Trichoderma*’s ability to inhibit *Fusarium* through myparasitism and secondary metabolites secretion. qRT-PCR analysis revealed a significant 9-fold overexpression of *lae1* in the OElae1 strain and a 3-fold overexpression of *vel1* in the OEvel1 strain, compared to the wild type (WT), indicating that *lae1* is a key factor in enhancing *Trichoderma* resistance to the pathogen (Figure S1). Furthermore, we evaluated the biomass of *Trichoderma* when cultured alone and in dual culture with pathogens, and the results showed that the biomass after confrontation was lower than after single grown (Figure 1C). Mlae1, Mvel1, and T23 had considerably decreased biomass compared to a single culture (*p* < 0.01), while OELae1 and OEvel1 demonstrated comparable development to single cultures. It is notable that the mycelial biomass of the Mvel1 mutant differed significantly from that of the wild-type T23 strain in both single and dual-culture assays. This implies a role for *vel1* in regulating hyphal growth and development, and confirms that this phenotype is consistent, even under biotic stress from the pathogen.

### 3.2. Total Peptaibols Production in Single Culture and In Vitro Trichoderma–F. graminearum Interaction

**Figure 2:**
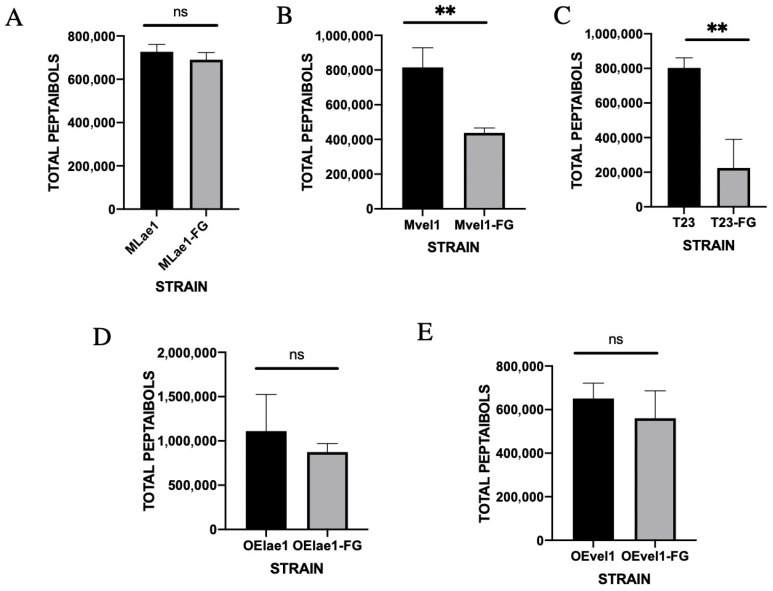
Changes in total peptide amount following confrontation between *Trichoderma* and its counterparts, *F. graminearum*, were assessed after normalization to single-culture controls: (**A**) Mlae1 vs. Mlae1-FG; (**B**) Mvel1 vs. Mvel1-FG; (**C**) T23 vs. T23-FG; (**D**) OElae1 vs. OElae1-FG s; (**E**) OEvel1 vs. OEvel1-FG. Error bars indicate SE of the mean of three replicates. Significant differences (** *p* < 0.01, ns, *p* > 0.5).

The peptaibols of the *Trichoderma* alone culture and *Trichoderma*–*F. graminearum* interaction culture were detected, and it can be observed that the peak time of peptaibols was 5–8 min from the ion flow diagram using UPLC-QTOF-MS (Figure 2A and Figure S2). The relative total amounts of peptaibols of the T23, OElae1, and OEvel1 strains showed a significant change when cultivated alone; OElae1 had an advantage, which is consistent with lae1. In comparison, the peptaibol amounts of the Mvel1 and T23 strains were much lower following confrontation than in a single culture (Figure 2B). The total peptaibols in T23 post-confrontation with *F. graminearum* dropped to approximately one-fourth of its initial level, whereas the total peptaibols content in Mvel1 diminished to half of its original quantity. The total peptaibols of the Mlae1, OElae1, and OEvel1 were not significantly different when compared to single cultures or in the presence of *F. graminearum*.

These findings indicated the profound influence of *Trichoderma*–*F. graminearum* interactions on the biosynthesis and secretion of peptaibols. In the case of Mvel1 and T23 confronted with *F. graminearum*, the overall biomass reduction in *Trichoderma*, stemming from nutritional and spatial competition, contributed to the observed decrease in total peptaibols detected. Conversely, OElae1 and OEvel1 strains maintained relatively stable biomass levels post-confrontation, aligning with the observed trends in peptaibol content. Intriguingly, despite a reduction in Mlae1 biomass subsequent to *F. graminearum* confrontation, the total peptaibol levels remained largely unchanged, suggesting a pathogen-induced upregulation of peptaibol secretion by Mlae1. These insights provide valuable perspectives on the intricate interplay between *Trichoderma* species and *F. graminearum*, with implications for biological control strategies.

### 3.3. Specific Peptaibol Production in Single Culture and In Vitro Trichoderma–F. graminearum Interactions

**Figure 3:**
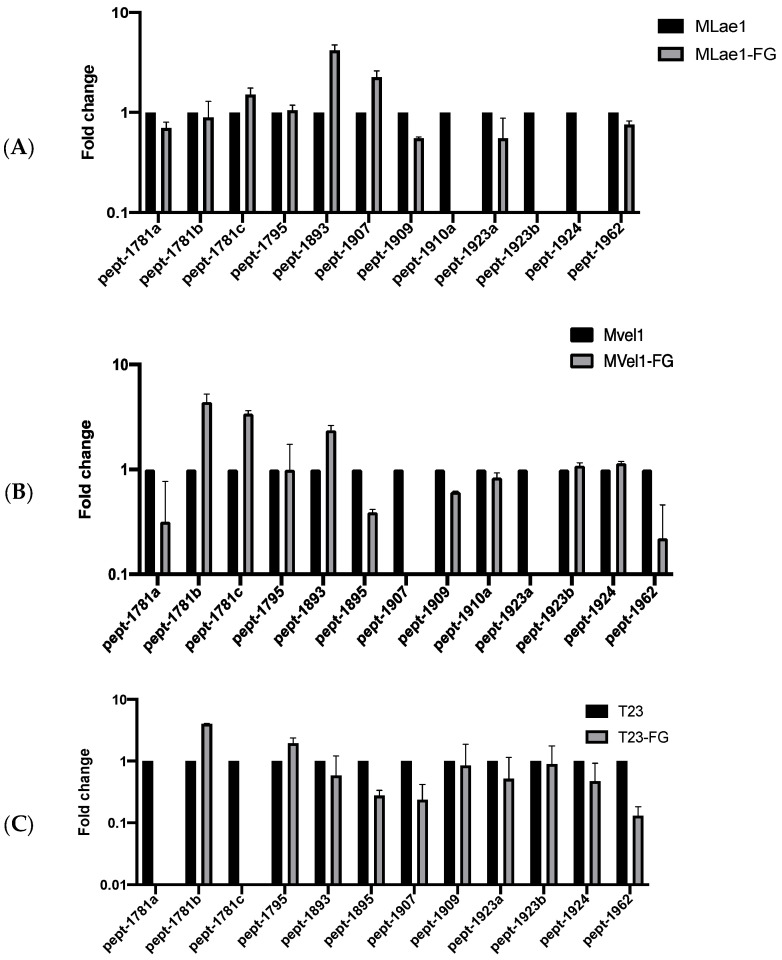

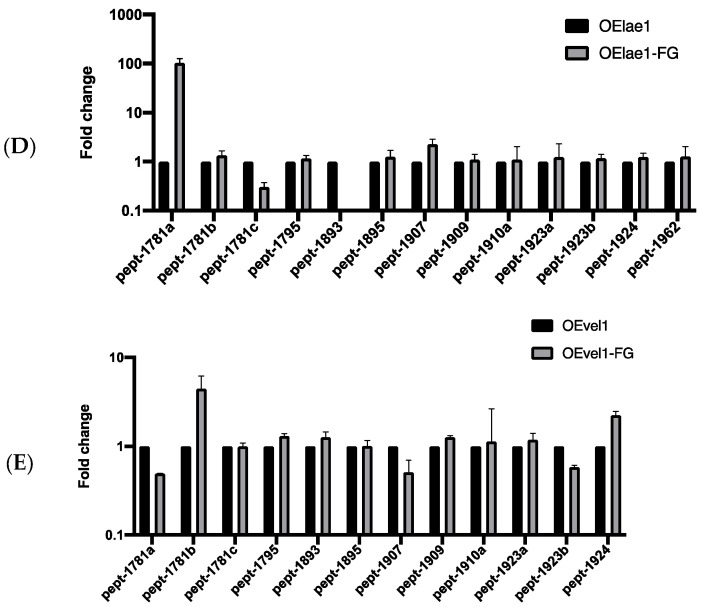
Comparison of relative peptaibol amounts in *Trichoderma* strains and their interactions with *F. graminearum* in confrontation tests: (**A**) Mlae1 vs. Mlae1-FG; (**B**) Mvel1 vs. Mvel1-FG; (**C**) T23 vs. T23-FG; (**D**) OElae1 vs. OElae1-FG s; (**E**) OEvel1 vs. OEvel1-FG.

Analyzing the mass spectrum in the chromatograms of peptaibols revealed that the wild-type strain T23 exhibited elevated levels of two peptaibols, pept-1781b and pept-1795, with pept-1781b increasing by a notable 4.03-fold, as shown in Figure 3 and Table S1. Similarly, the Mlae1 strain demonstrated increased contents of pept-1781c, pept-1893, and pept-1907 post-confrontation, particularly pept-1893, which surged 4.18-fold. In Mvel1, pept-1781b, pept-1781c, and pept-1893 showed marked enhancements of 4.40, 3.43, and 4.40 times, respectively. OEvel1 also displayed augmented levels of pept-1781b, pept-1795, pept-1909, and pept-1924, with pept-1781b increasing by 4.44-fold. Notably, the OElae1 strain exhibited a remarkable, nearly 100-fold increase in pept-1781a content upon confrontation.

These findings indicated that the overexpression strains OElae1 confronted with *F. graminearum* exhibited an enhanced diversity and quantity of peptaibols, conferring them with superior antagonistic properties against the pathogen. Notably, pept-1781b and pept-1781a were each significantly upregulated in OElae1and OEvel1 strains, suggesting they were positively regulated by LAE1 and VEL1 transcription factors. However, the response to *F. graminearum* confrontation displayed contrasting trends, with Pept-1781a overexpressed exclusively in OElae1 (100-fold), while undetectable in T23, indicating its induction is dependent on the Lae1. Conversely, PEPt-1781b increased across strains Mvel1, T23, OEvel1, and OElae1 to varying degrees, suggesting its expression is *F. graminearum*-induced and independent of Velvet proteins. Intriguingly, T23 failed to produce PEPT-1781c upon confrontation, while LAE1 and VEL1 negatively regulated these peptaibols, suggesting that variations in amino acid stereochemistry may account for this case. Taken together, the presence of *F. graminearum* triggered the secretion of 18-residue peptaibols, specifically pept-1781b and pept-1781a, while suppressing the synthesis of pept-1781c. The former two peptaibols are also induced by *lae1* and *vel1* overexpression and promote pept-1781b and pept-1781a secretion.

### 3.4. Analysis of Peptaibols Co-Induced by F. graminearum and Velvet Protein

Analysis of the MS chromatograms of crude extracts from control and confronted cultures revealed five distinct peptaibols were identified: Pept-1781a, Pept-1781b, Pept-1781c, Pept-1795, and Pept-1893, as shown in Table 1. The tandem mass spectra graph for the main peptaibols is shown in Figure S3. These peptaibols exhibited some similarities to Trichorzins PA, Trichorzin TVB I, and Trichorzin TVB II, with the notable exception of their amino acid composition at the second position in detail. Notably, Pept-1781c, Pept-1781a, and Pept-1781b, all being 18-residue peptaibols, were in possession of significantly varied amino acid sequences. Specifically, the 3rd, 6th, 7th, 9th, and 11th amino acid positions in PEPT-1781a differ markedly from those in Pept-1781b and Pept-1781c, contributing to the formation of distinct peptaibol structures. The curved secondary structure of these peptaibols was determined by the 18 residues located at the R11-R13 position of the Aib-Pro bond. Notably, glutamine (Gln) at position 6 plays a pivotal role in forming cell membrane ion channels, whereas leucine-xx (Lxx) at position 6 in Pept-1781a substitutes for Gln. Furthermore, alanine (Ala) at position 3, glycine (Gly) at position 9, and asparagine (Asn) and aspartic acid (Asp) at positions 10 and 11, respectively, differ from the other two peptaibols. The frequency and position of Aib in each peptaibol indicate the formation of helical structures, including α-helices or 3,10-helices, though whether these secondary structures facilitate helical or ion channel formation remains an open question.

Regulation of Pept-1781a, Pept-1781, and Pept-1781c was governed by both *F. graminearum* and velvet transcription factors. The disparity in their amino acid sequences impacts their secondary structures, with all three contributing to *Trichoderma*’s inhibition of *F. graminearum* through differential secretion. Notably, overexpression of *lae1* and *vel1* strains significantly boosted the production of Pept-1781a and Pept-1781b, thereby enhancing their synergistic effect.

### 3.5. Effects of Crude Peptaibol Extract on the Growth of F. graminearum

**Figure 4:**
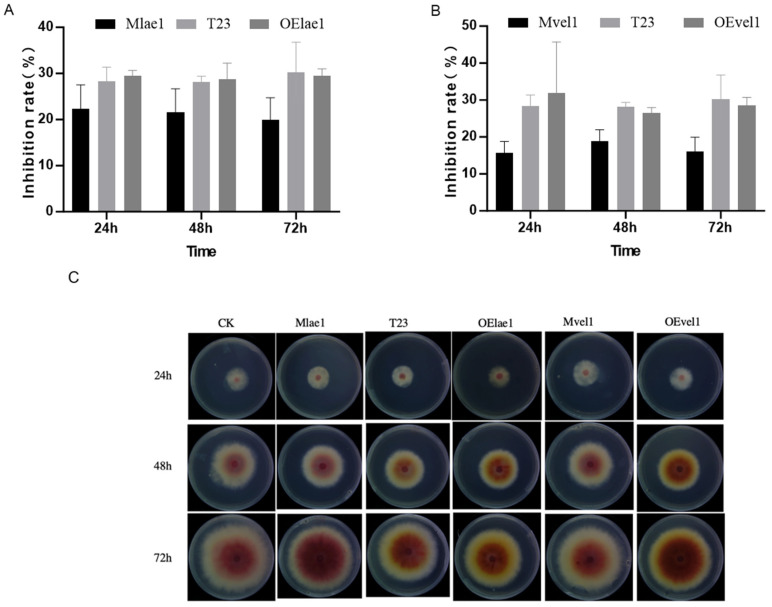
Effect and inhibition rate (%) of peptaibol crude extracts from *Trichoderma* strain T23 and its counterpart against *F. graminearum*: (**A**) Inhibition rate of Mlae1, T23 and OElae1; (**B**) inhibition rate of Mvel1, T23 and OEvel1; (**C**) the growth dynamics alteration of *F. graminearum* were monitored from 24 h to 72 h in the presence of peptaibol crude extracts.

Inhibition rates of the peptaibols extracts from different *Trichoderma* strains were compared, as Figure 4A shows the antifungal rates of Mlae1, T23, and OElae1 strains against *F. graminearum*. No statistically significant differences in antifungal activity against *F. graminearum* were observed for crude extracts across the time-course dynamic (one-way ANOVA, *p* > 0.05). Strikingly, the OElae1 strain demonstrated a 1.5-fold higher inhibition rate compared to the Mlae1 mutant (Student’s *t*-test, *p* < 0.01). Similarly, the OEvel1 exhibited twice the antifungal efficacy of its corresponding Mvel1 strain (*p* < 0.001), highlighting the dosage-dependent regulatory role of Velvet components in biocontrol potency. The results demonstrated that knockout of *lae1* and *vel1* significantly decreased antifungal activity against *F. graminearum,* revealing a positive regulatory role for velvet components in the synthesis of antifungal metabolites. Notably, *lae1* emerged as the dominant regulator, contributing 68% of the variance in peptaibol yields, suggesting its central role in orchestrating this metabolic pathway.

### 3.6. Effects of Crude Peptaibol Extracts on the Membrane Permeability of F. graminearum

The electrical conductivity assay was utilized to evaluate the impact of crude peptaibol extracts on the membrane permeability of *F. graminearum*.

**Figure 5:**
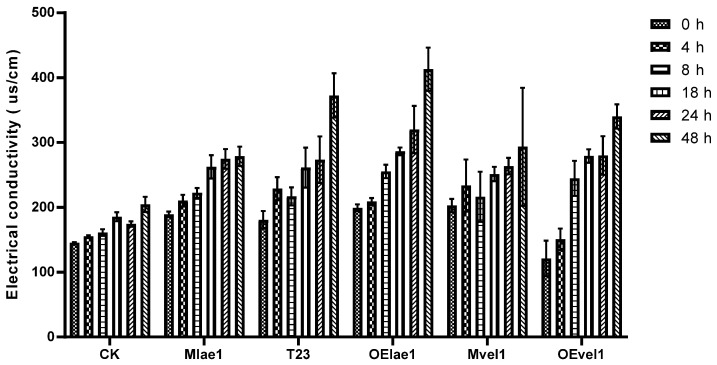
Changes in electrical conductivity of *F. graminearum* plasma membranes treated with crude peptaibol extracts from *Trichoderma* and its derivatives (CK, T23, Mlae1, OElae1, Mvel1, and Ovel1).

A higher electrical conductivity indicates a stronger binding effect between the crude peptaibols extracts and the cell membrane. As depicted in Figure 5, the electrical conductivity of CK (control) remained relatively unchanged at 0 h, 4 h, 8 h, 18 h, 24 h, and 48 h, whereas significant changes were observed in the treatments with crude extracts from T23, OElae1, and OEvel1 strains. Specifically, the electrical conductivity of samples treated with the crude extract from the OElae1 strain increased from 199.3 μs/cm to 413 μs/cm, representing a 107.2% increase. Similarly, the electrical conductivity of samples treated with the OEvel1 strain crude extract rose from 121 μs/cm to 340 μs/cm, with a nearly 3-fold increase. In contrast, the electrical conductivity of CK only increased from 145.4 μs/cm to 204.5 μs/cm. These results showed that the overexpression of *lae1* or *vel1* enhances the membrane permeability of *F. graminearum*, leading to increased vulnerability and reduced survival rates for the pathogen.

### 3.7. Effects of Crude Peptaibol Extracts on the Production of Deoxynivalenol by F. graminearum

**Figure 6:**
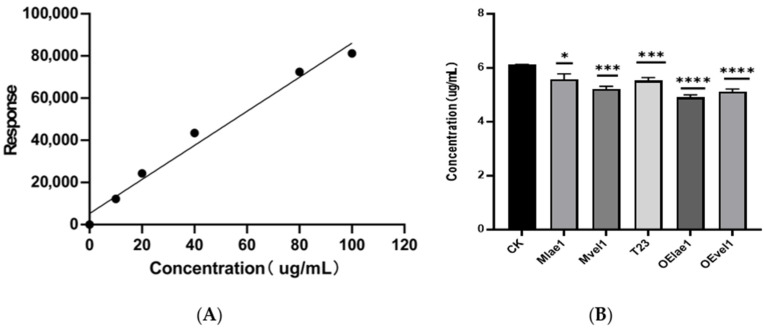
Comparison of the DON concentration of *F. graminearum* treated with crude extracts from five *Trichoderma* strains (Mlae1, Mvel1, T23, OElae1, and OEvel1): (**A**) The standard curve was generated by plotting the mass spectrometry values against known concentrations of DON; (**B**) concentration of DON in *F. graminearum*. Error bars indicate SE of the mean of three replicates. Significant differences (**** *p* < 0.0001, *** *p* < 0.001, * *p* < 0.05).

Using the concentration of (DON) as the horizontal axis and the response value in the mass spectrometry as the vertical axis, a standard curve was plotted for DON, as shown in Figure 6A. The standard curve is y = 878.9x (R^2^ = 0.996), allowing for the quantification of vomitoxin content in both the peptaibols-treated group and the control group through this curve. The concentration of DON in *F. graminearum* cultures treated with peptaibol crude extracts from five different *Trichoderma* strains (Mlae1, Mvel1, T23, OElae1, and OEvel1) was measured to assess the efficacy of these extracts in reducing toxin production. As depicted in Figure 6B, the production of DON by *F. graminearum* was significantly reduced after different treatments, exhibiting a notable difference compared to the control group (*p* < 0.05). Notably, the crude extract of peptaibols from OElae1 treated *F. graminearum* produced the lowest level of DON with 4.97 μg/mL, followed by the OEvel1 crude extract treatment with 5.14 μg/mL, which showed a decrease of 23.14% and 19.07%, respectively, compared to the control (6.12 μg/mL). This result underscored the potential of crude extracts in reducing DON production in *F. graminearum*, with the crude extracts from the overexpression *lae1* and *vel1* strains being particularly effective in mitigating DON production.

## 4. Discussion

*Trichoderma* species are prolific producers of diverse secondary metabolites, including peptaibols, which play critical roles in microbial antagonism and plant immunity. In this study, we provide the first mechanistic insights into peptaibol biosynthesis dynamics during in vivo antagonism between *Trichoderma* and *Fusarium graminearum*. Time-resolved metabolic profiling revealed significant perturbations in peptaibol abundance and diversity during fungal confrontations, with strain-specific responses highlighting the regulatory roles of *lae1* and *vel1*. Of note, overexpression of *lae1* and *vel1* in *Trichoderma* strains (OElae1/OEvel1) resulted in a significant fold increase in specific peptaibol production (*p* < 0.01), accompanied by enhanced antagonistic efficacy against *F. graminearum*. In contrast, Mvel1 and Mlae1 mutants showed reduced peptaibol yields and compromised biocontrol performance, with Mvel1 exhibiting a 70% reduction in total peptaibol content during confrontation. These results underscore that the synthesis of peptaibols could be induced by interacting with the pathogen and regulated mediate velvet complex, amplifying both the quantity and diversity of peptaibols, offering a potential approach for peptaibol production.

### 4.1. Strain-Specific Peptaibol Dynamics Synthesis Regulated via Velvet Complex

The observed decrease in total peptaibol production after confrontation, combined with the specific induction of certain peptaibols by *lae1* and *vel1* in the presence of *Fusarium graminearum* (potentially as a targeted response), indicates a survival strategy under stress. This modulation not only enhanced the quantity of effective antifungal metabolites but also generated novel peptaibols, thereby bolstering biocontrol efficacy. Similar to these findings, Tamandegani et al. revealed an increase in the total peptaibol production during interactions with six different pathogens, as well as differences in peptaibol profiles between confrontational and control tests [37].

Distinct peptaibol modulation patterns were observed across *Trichoderma* strains during *F. graminearum* confrontation. While T23 and Mvel1 exhibited parallel declines in biomass and peptaibol content, Mlae1 maintained stable peptaibol levels despite biomass reduction, suggesting pathogen-induced biosynthesis activation. Notably, *F. graminearum* selectively induced 18-residue peptaibols (e.g., Pept-1781a/b/c, Pept-1795) as 18-residue variants sharing functional similarities with characterized peptaibols like neoatroviridins and Trichorzins PA, which disrupted membrane integrity and prime plant defense pathways [41]. *T. longibrachiatum* strain 40418 produces two peptaibols, trilongin AIV a (11-residue) and trilongin BI (20-residue), which significantly induce plant resistance to *Pseudomonas syringae* pv. tomato DC3000 infection and triggers plant immunity and cell death [42].

In addition to inhibiting pathogens and inducing plant resistance, recent studies have shown that peptaibols in *Trichoderma* can inhibit the development of clinical *Staphylococcus aureus* infections and cancer cells, indicating greater potential for clinical applications. Seven new 18-residue peptaibols, trichorzins A-G (1–7) and Trichorzin PA, were isolated from the sponge-derived fungus *Trichoderma sp*. GXIMD 01001 and *T. lentiforme* ML-P8-2, which not only show potent antibacterial activity but also exhibit significant cytotoxicity against human cancer cell lines as well [43,44]. The 18-AA peptaibols in *T. guizhouense* were found to exhibit cytotoxicity against MDA-MB-231, SK-Hep1, SKOV3, DU145, and HCT116 cells greater than that of the 14-AA peptaibols [45]. The induction of specific peptaibols by both global regulators and pathogen signals highlighted a sophisticated regulatory network that tailors metabolite production to environmental challenges. Studies have demonstrated that Tlstp1, a glucose sensor orthologue in *T. longibrachiatum* SMF2, significantly regulates peptaibol production by influencing the transcription of N NRPS genes *tlx1* and *tlx2*. Knockout of the glucose sensor Tlstp1 increased Trichokonin yields by up to 5-fold via transcriptional activation of NRPS genes, revealing a genetic target for engineering over-producing strains [46].

### 4.2. Peptaibols Increase Cell Membrane Permeability and Regulate DON Biosynthesis in Fusarium graminearum

The membrane plays a crucial role in maintaining the internal balance of the cell, including the regulation of ion concentrations and the transport of nutrients and waste products. Increased permeability can disrupt this homeostasis, leading to imbalances that can impair cellular functions and potentially cause cell death. Due to their amphipathic nature, peptaibols can insert into lipid bilayer membranes and assemble into voltage-dependent ion channels [47]. The bactericidal effect of 18-residue peptaibol trichorzins PA on mycoplasmas is due to increased membrane permeabilization, independent of cholesterol content, as is the case with the pore-forming peptaibols saturnisporin SA IV and harzianin HA V [41,48]. We also observed that enhanced peptaibols increased the pathogen’s membrane permeability, thereby suppressing its viability. Crude extracts from these strains exhibited multifunctional antifungal activity, including suppression of hyphal extension, increased membrane permeability, and inhibition of deoxynivalenol (DON) biosynthesis.

In addition, as the main mycotoxin of *F. graminearum* infecting wheat and other plants, DON easily exists in various cereal grains and processed products, posing a hazard to both plants and humans. Besides being capable of controlling phytopathogens, some *Trichoderma* strains have the potential to metabolize hazardous contaminants. Tian et al. found that *Trichoderma* spp. were capable of glycosylating type A trichothecenes into glycosylated forms against mycotoxin and self-production [49]. The effects of velvet-mediated peptaibols on mycotoxin synthesis have not been adequately investigated. Our present study suggests that *Trichoderma*-derived crude extracts significantly inhibit the production of deoxynivalenol (DON), the primary trichothecene mycotoxin responsible for *F. graminearum*’s virulence in cereal crops. The extracts used for bioassays consisted predominantly of peptaibols, as indicated by the UPLC-MS/MS data, despite the presence of other metabolites in the crude extracts. The reduction in DON accumulation observed here is likely linked to peptaibol-mediated membrane permeabilization, which disrupts the energy-dependent metabolic flux and enzymatic activities essential for trichothecene biosynthesis. Similarly, lipopeptides (LPs) such as iturin and fengycin from *Bacillus amyloliquefaciens* S76-3 strongly inhibit pathogenic *F. graminearum*. This is due to LPs causing cell swelling by triggering cell wall remodeling and glycerol synthesis via cell wall integrity, and reducing deoxynivalenol accumulation [50].

Our study suggests that velvet-mediated regulation of peptaibols might involve the inhibition of DON synthesis through three possible pathways: (i) direct membrane permeabilization compromises both energy supply and toxin efflux; (ii) interference with global regulators such as *vel1* and *lae1* and further downregulates *Tri* gene expression; and (iii) metabolic reallocation further limits precursor flux into the trichothecene pathway. Together, these mechanisms position peptaibols as dual-function agents that not only inhibit pathogen growth but also prevent its mycotoxin arsenal. This dual action of enhancing cell membrane permeability and reducing mycotoxin accumulation makes peptaibols a promising candidate for the development of new strategies to control fungal diseases and reduce the associated risks posed by mycotoxins in agricultural and post-harvest food safety contexts.

### 4.3. Enhancing Peptaibol-Based Biocontrol Effective Through lae1-Mediated Regulation

As we know, the fast growth of *Trichoderma* confers a competitive advantage in dual-culture assays, enabling overgrowth and inhibition of *F. graminearum*. Our results showed that the inhibition rate in dual culture was significantly higher than that of the crude extracts alone, indicating the combined effects of mycoparasitism and antifungal metabolites from Trichoderma. The enhanced antifungal activity of the OElae1 crude extract suggests a potential increase in the production of inhibitory compounds, such as peptaibols. While the presence of other antifungal compounds in the crude extract cannot be ruled out, future research should focus on isolating and characterizing individual peptaibols to elucidate their mode of action and better understand the contribution of peptaibols against the pathogen.

The application of cocktails combining enzymes and diverse secondary metabolites, such as peptaibols, from *Trichoderma* holds promise for the future of biocontrol. To enhance the efficacy of these peptaibols, strategies involving genetic engineering and fermentation optimization can be employed. This study identifies two key engineering targets for enhancing *Trichoderma*’s biocontrol potential: (1) induction of *Fusarium* to enhance the secretion of targeted antimicrobial peptaibols; (2) manipulating *lae1* regulators to expand peptaibols output. Increased yields would allow for peptaibol purification, facilitating both mechanistic studies and the development of peptaibol-based biocontrol strategies. By leveraging velvet-mediated regulatory networks, it is possible to design peptaibol-hyperproducing *Trichoderma* strains with enhanced biocontrol efficacy, developing next-generation bio-fungicides that exploit Velvet-regulated antifungal arsenals. Our work bridges fungal molecular ecology and synthetic biology, offering a roadmap to reprogram microbial warfare through master transcriptional hubs.

## 5. Conclusions

This study elucidates the pivotal role of velvet-mediated regulatory networks in modulating peptaibol biosynthesis, thereby facilitating molecular crosstalk between *Trichoderma* and the phytopathogen *Fusarium graminearum*. By examining the functions of *lae1* and *vel1*, we demonstrate that manipulating the *lae1* regulator can significantly enhance *Trichoderma*’s biocontrol efficacy against *F. graminearum*. Our findings highlight that *Trichoderma* species utilize dynamic peptaibol modulation as a strategic defense mechanism, involving both the upregulation of long-residue peptaibols and the synthesis of novel structural variants, which likely act on the cell membrane and affect DON production indirectly. This research not only provides insights into the adaptive responses of *Trichoderma* to pathogen infection but also opens new avenues for developing broad-spectrum antifungal agents and identifying plant growth regulators for agricultural applications.

## Figures and Tables

**Table 1:** Sequence of main peptaibols in *Trichoderma* strains during confrontation with *F. graminearum*.

Peptaibols	1	2	3	4	5	6	7	8	9	10	11	12	13	14	15	16	17	18	19
Pept-1781a	AcAib	Ala	Ala	Aib	Vxx	Lxx	Vxx	Aib	Gly	Asn	Asp	Aib	Pro	Lxx	Aib	Aib	Gln	Pheol	
Pept-1781b	AcAib	Ala	Aib	Aib	Aib	Gln	Aib	Aib	Aib	Ser	Lxx	Aib	Pro	Lxx	Aib	Aib	Gln	Pheol	
Pept-1781c	AcAib	Ala	Aib	Aib	Vxx	Gln	Aib	Ala	Aib	Ser	Lxx	Aib	Pro	Lxx	Aib	Aib	Gln	Pheol	
Pept-1795	AcAib	Ala	Aib	Aib	Vxx	Gln	Aib	Aib	Aib	Ser	Lxx	Aib	Pro	Lxx	Aib	Aib	Gln	Pheol	
Pept-1893	AcAib	Ala	Aib	Aib	Aib	Gln	Aib	Aib	Aib	Ala	Lxx	Aib	Pro	Lxx	Aib	Aib	Gln	Gln	Pheol
Trichorzin TVB I	AcAib	Gly	Ala	Val	Aib	Gln	Aib	Ala	Aib	Ser	Leu	Aib	Pro	Leu	Aib	Aib	Gln	Valol	
Trichorzin TVB II	AcAib	Gly	Ala	Leu	Aib	Gln	Aib	Ala	Aib	Ser	Leu	Aib	Pro	Leu	Aib	Aib	Gln	Valol	
Trichorzin PA II	AcAib	Ser	Ala	Aib	Iva	Gln	Aib	Val	Aib	Gly	Leu	Aib	Pro	Leu	Aib	Aib	Gln	Trpol	
Trichorzin PA IV	AcAib	Ser	Ala	Aib	Iva	Gln	Iva	Val	Aib	Gly	Leu	Aib	Pro	Leu	Aib	Aib	Gln	Trpol	
Trichorzin PA IX	AcAib	Ser	Ala	Iva	Iva	Gln	Aib	Val	Aib	Gly	Leu	Aib	Pro	Leu	Aib	Aib	Gln	Pheol	
Trichorzin PA V	AcAib	Ser	Ala	Iva	Iva	Gln	Aib	Val	Aib	Gly	Leu	Aib	Pro	Leu	Aib	Aib	Gln	Trpol	
Trichorzin PA VI	AcAib	Ser	Ala	Aib	Iva	Gln	Aib	Val	Aib	Gly	Leu	Aib	Pro	Leu	Aib	Aib	Gln	Pheol	
Trichorzin PA VII	AcAib	Ser	Ala	Iva	Iva	Gln	Iva	Val	Aib	Gly	Leu	Aib	Pro	Leu	Aib	Aib	Gln	Trpol	
Trichorzin PA VIII	AcAib	Ser	Ala	Aib	Iva	Gln	Iva	Val	Aib	Gly	Leu	Aib	Pro	Leu	Aib	Aib	Gln	Pheol	

## Data Availability

The original contributions presented in this study are included in the article/Supplementary Material. Further inquiries can be directed to the corresponding author.

## Supplementary Materials

The following supporting information can be downloaded at: https://www.mdpi.com/article/10.3390/microorganisms14040847/s1, Figure S1: qRT-PCR analysis of relative expression level of *vel1* and *lae1* in over-expression (OEvel1 and OElae1) and wild type T23. Figure S2: Ion Flow diagram of *Trichoderma* (alone) and *Trichoderma* towards *F. graminearum* (Fg) A: Mlae1 and Mlae1 vs. Fg; B: Mvel1 and Mvel1 vs. Fg; C: T23 and T23 vs. Fg; D: OElae1 and OElae1 vs. Fg strain; E: OEvel1 and OEvel1 vs. Fg. Figure S3: MS/MS Spectra of Selected Peptaibols Acquired by UPLC-QTOF-MS/MS. Table S1: Molecular characteristics and fold change of difference peptaibols in five comparative groups (Mlae1 vs. Mlae1-FG, Mvel1 vs. Mvel1-FG, T23 and T23-FG, OElae1 vs. OElae1-FG, OEVel1 vs. OEvel1-FG).
