# Supplementary material for: Regulation of Peptaibol Profile by Velvet LAE1/VEL1 in Trichoderma Species During In Vitro Confrontations with Fusarium graminearum
Source: Microorganisms. 2026 Apr 9;14(4):847. doi: 10.3390/microorganisms14040847 (PMC13119502; doi:10.3390/microorganisms14040847)
Supplement: Supplementary file 1 [file microorganisms-14-00847-s001.zip › microorganisms-4182411-supplementary/Supplemental files_Figure S1-S3.pdf]

Figure. S1 qRT-PCR analysis of relative expression level of *vel1* and *lae1* in over-expression (OEvel1 and OElae1) and wild type T23.

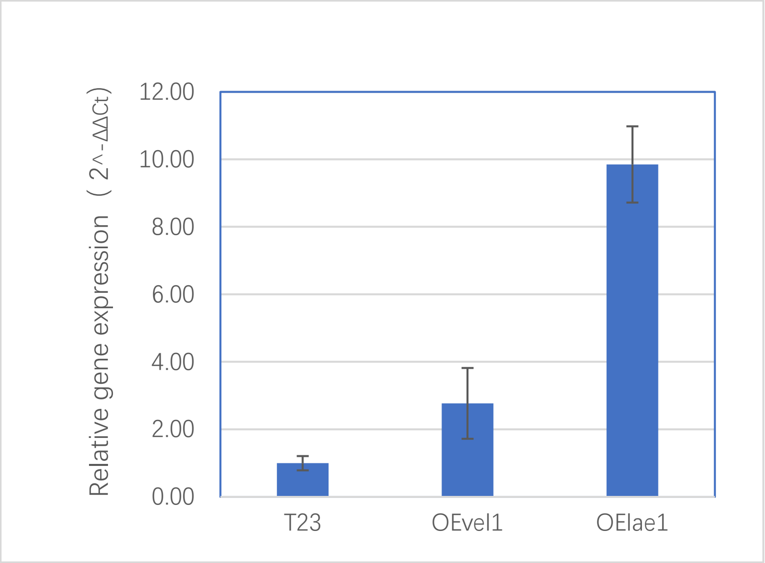

Figure. S2 Ion Flow diagram of *Trichoderma* (alone) and *Trichoderma* towards *F.graminearum* (Fg) A: Mlae1 and Mlae1 vs Fg ; B: Mvel1 and Mvel1 vs Fg; C: T23 and T23 vs Fg; D: OElae1 and OElae1 vs Fg strain; E: OEvel1 and OEvel1 vs Fg

A

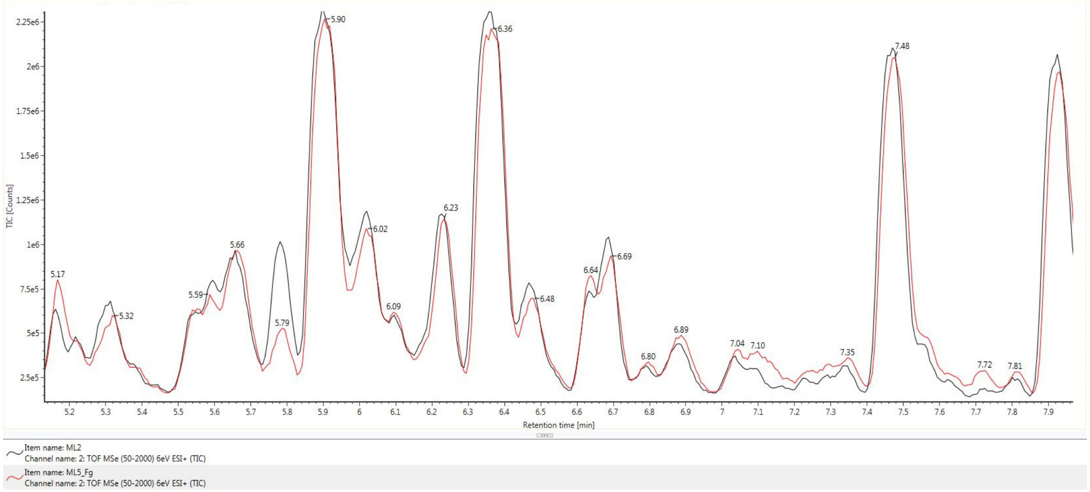

B

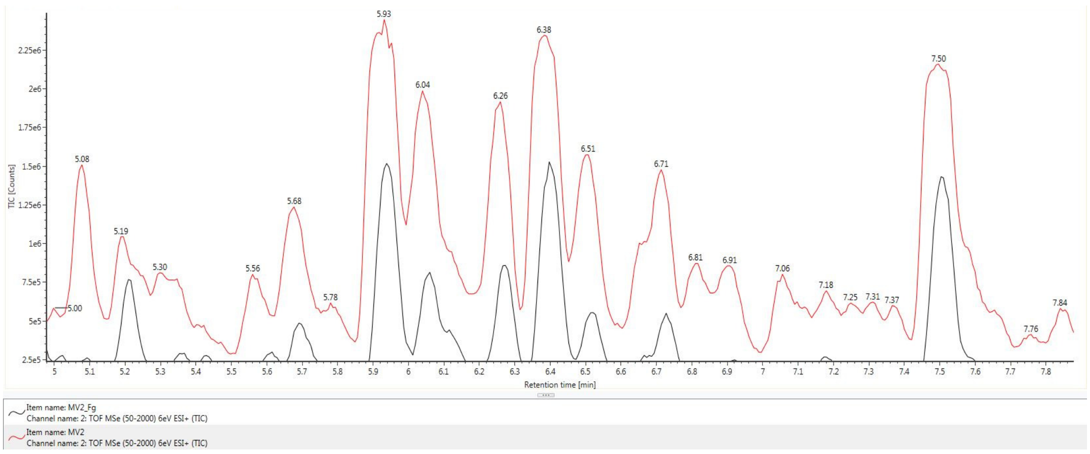

C

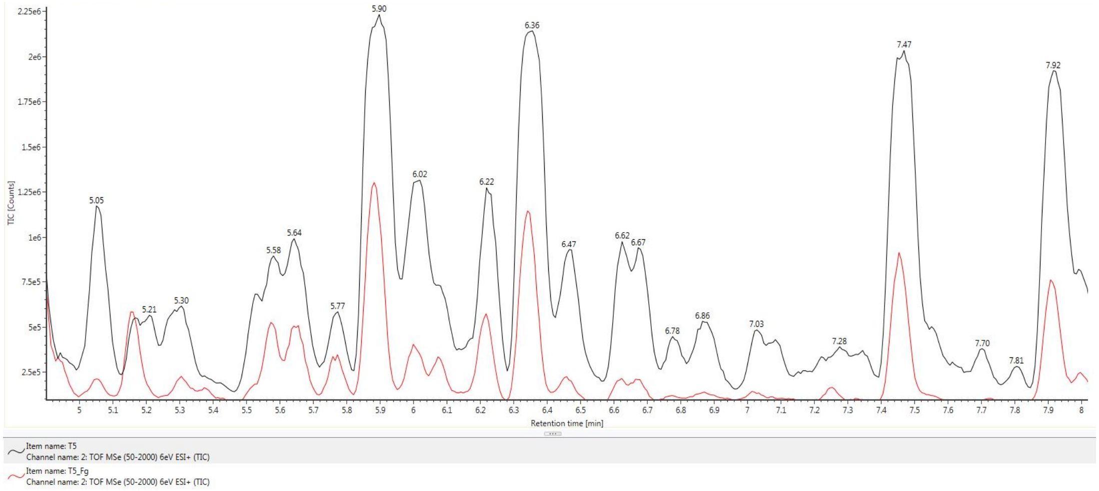
TIC [Counts]
Retention time [min]
Item name: T5
Channel name: 2: TOF MSe (50-2000) 6eV ESI+ (TIC)
Item name: T5_Fg
Channel name: 2: TOF MSe (50-2000) 6eV ESI+ (TIC)

D

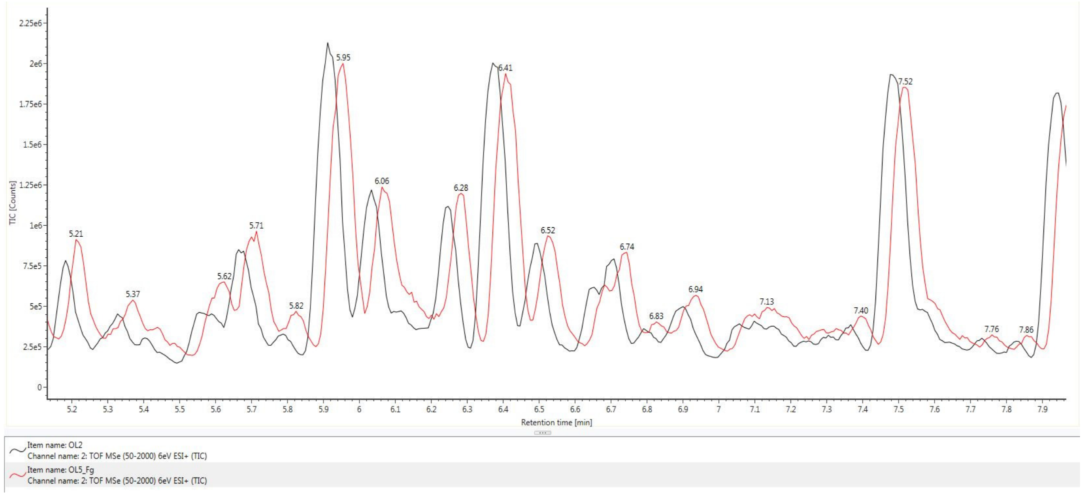
TIC [Counts]
Retention time [min]
Item name: OL2
Channel name: 2: TOF MSe (50-2000) 6eV ESI+ (TIC)
Item name: OL5_Fg
Channel name: 2: TOF MSe (50-2000) 6eV ESI+ (TIC)

E

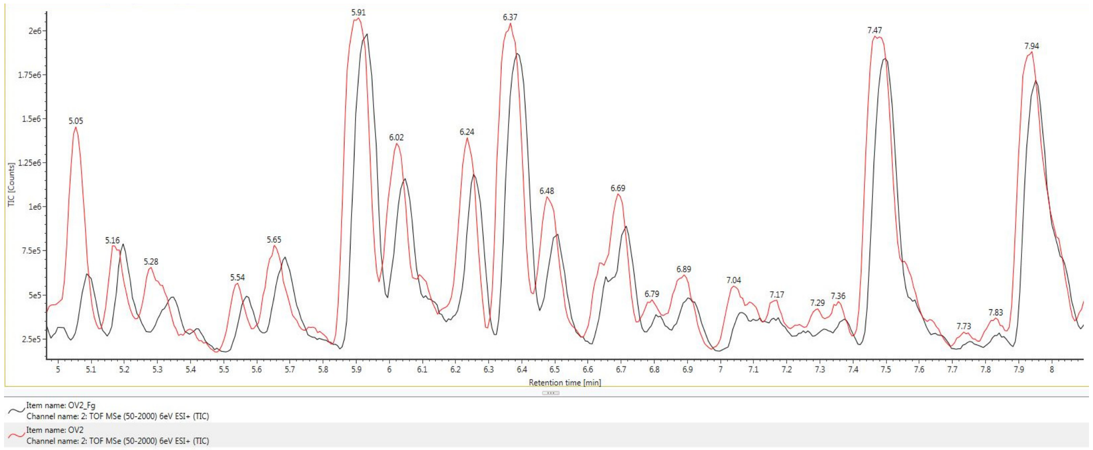
TIC [Counts]
Retention time [min]
Item name: OV2_Fg
Channel name: 2: TOF MSe (50-2000) 6eV ESI+ (TIC)
Item name: OV2
Channel name: 2: TOF MSe (50-2000) 6eV ESI+ (TIC)

Figure S3 MS/MS Spectra of Selected Peptaibols Acquired by UPLC-QTOF-MS/MS

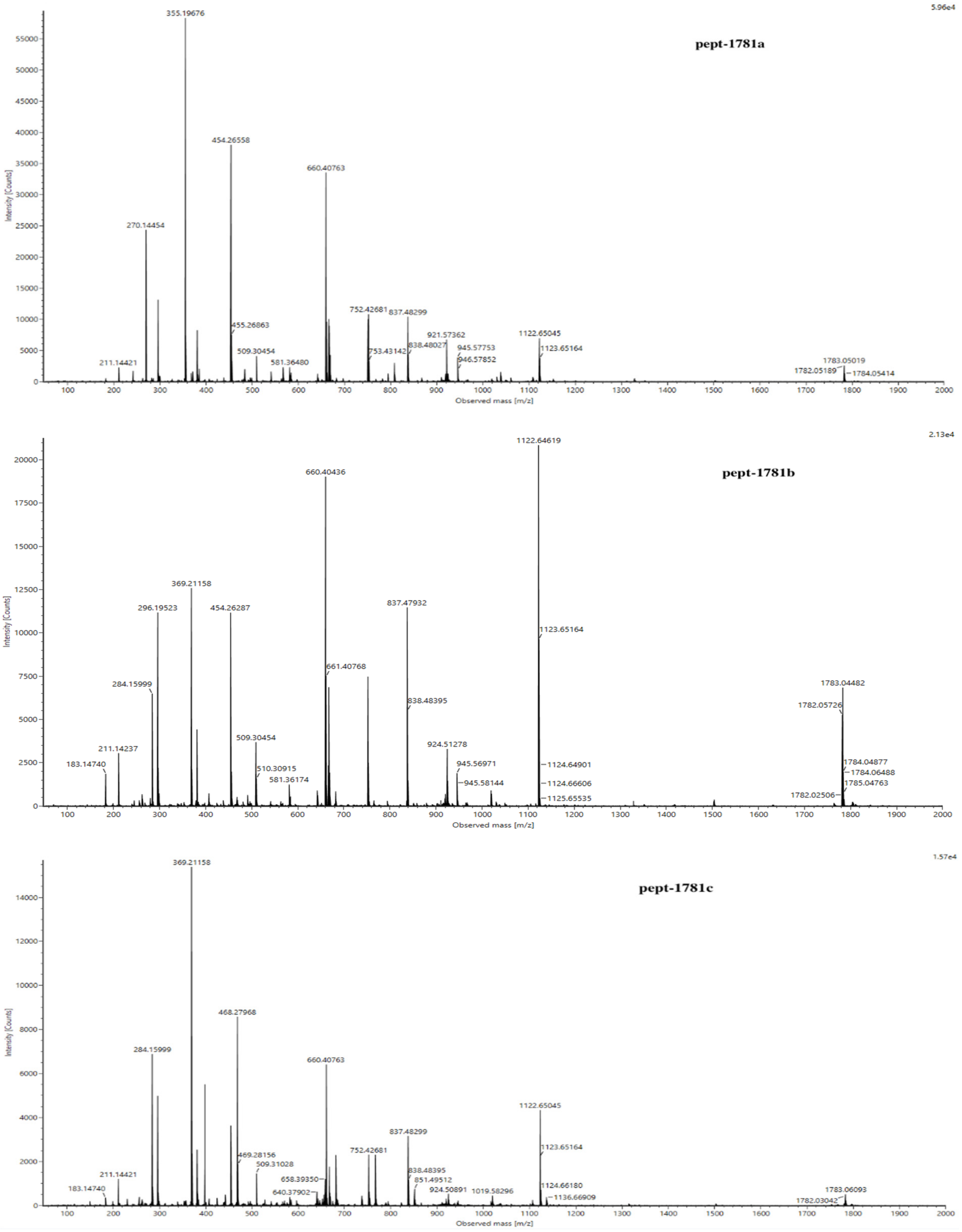

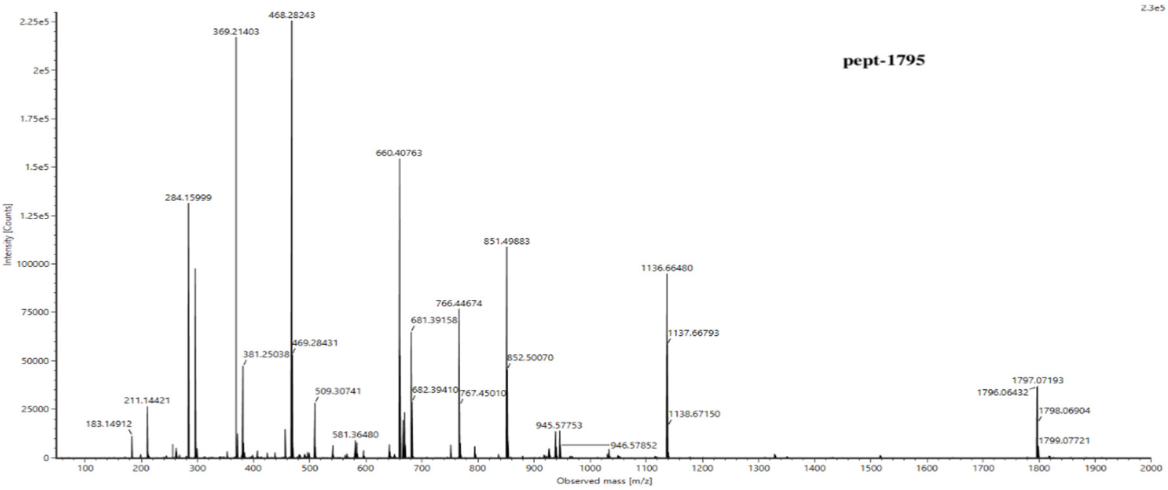
pept-1795
2.3e5
Intensity [Counts]
Observed mass [m/z]
468.28243
369.21403
660.40763
284.15999
851.49883
1136.66480
766.44674
681.39158
1137.66793
469.28431
381.25038
852.50070
1797.07193
1796.06432
211.14421
509.30741
682.39410
767.45010
1798.06904
183.14912
1138.67150
945.57753
581.36480
946.57852
1799.07721

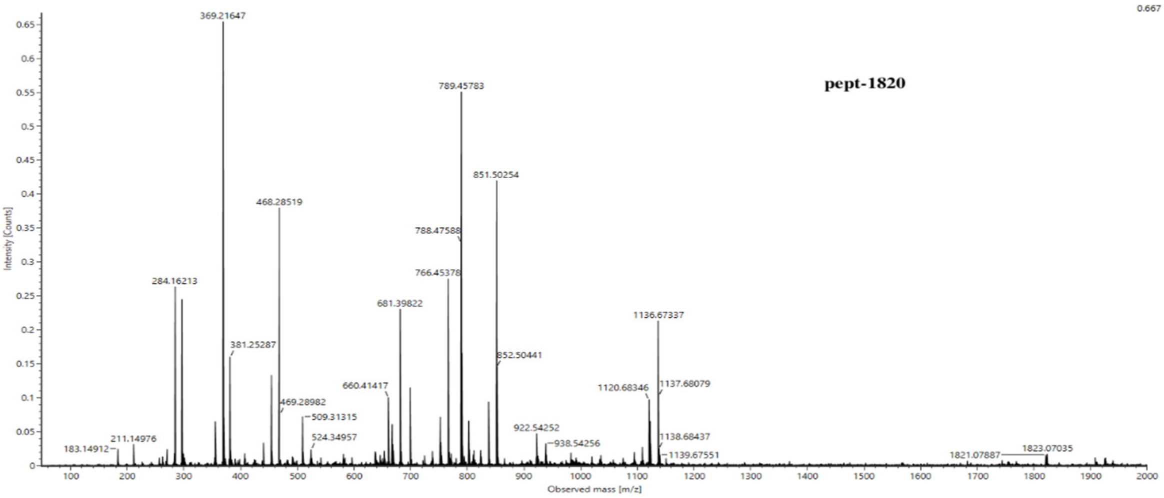
pept-1820
0.667
Intensity [Counts]
Observed mass [m/z]
369.21647
789.45783
851.50254
468.28519
788.47588
766.45378
284.16213
681.39822
1136.67337
381.25287
852.50441
660.41417
1120.68346
1137.68079
469.28982
509.31315
922.54252
211.14976
524.34957
938.54256
1138.68437
183.14912
1139.67551
1821.07887
1823.07035

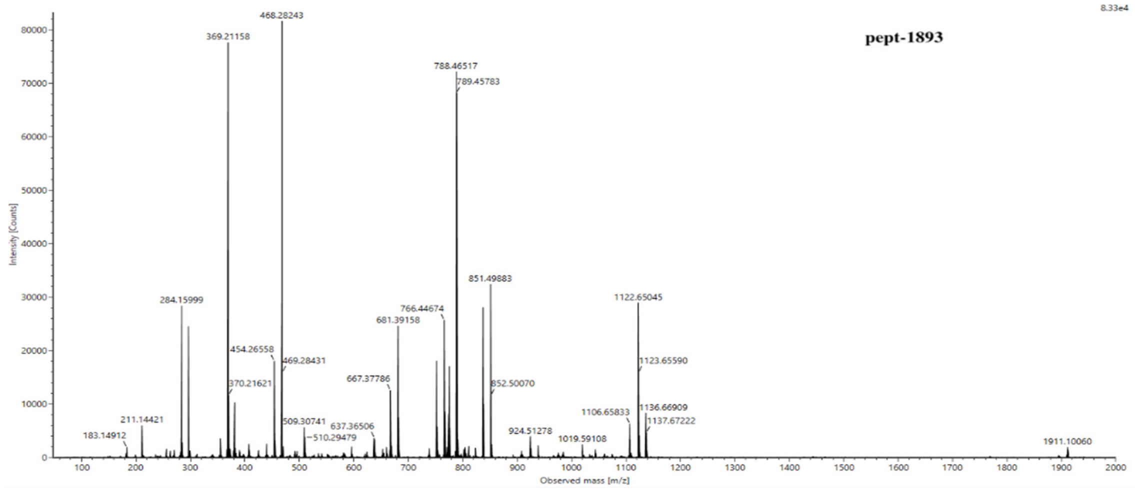
pept-1893
8.33e4
Intensity [Counts]
Observed mass [m/z]
468.28243
369.21158
788.46517
789.45783
851.49883
284.15999
1122.65045
766.44674
681.39158
454.26558
469.28431
1123.65590
667.37786
370.21621
852.50070
1106.65833
1136.66909
211.14421
509.30741
637.36506
1137.67222
924.51278
183.14912
510.29479
1019.59108
1911.10060

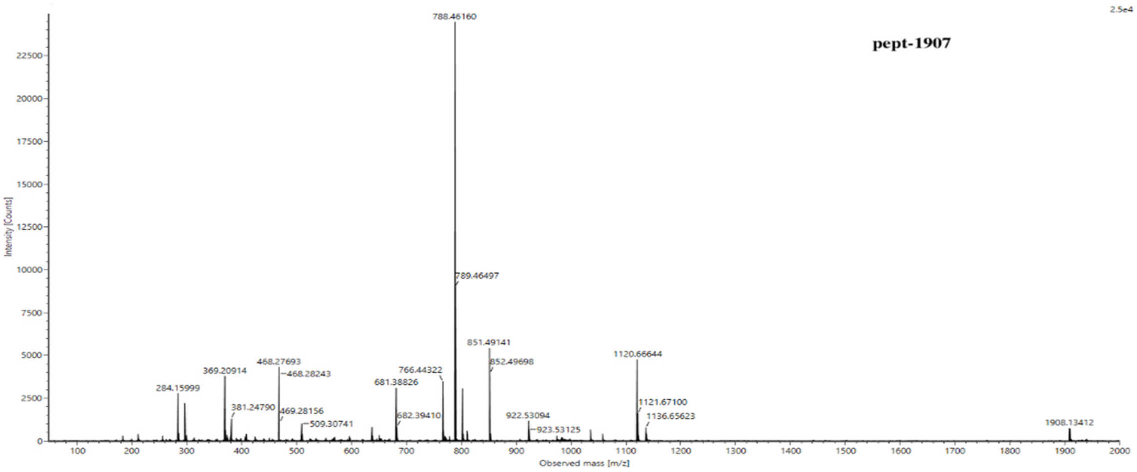
pept-1907
2.5e4
Intensity [Counts]
Observed mass [m/z]
788.46160
789.46497
851.49141
852.49698
1120.66644
1121.67100
1136.65623
766.44322
681.38826
682.39410
468.27693
468.28243
469.28156
509.30741
369.20914
381.24790
284.15999
922.53094
923.53125
1908.13412

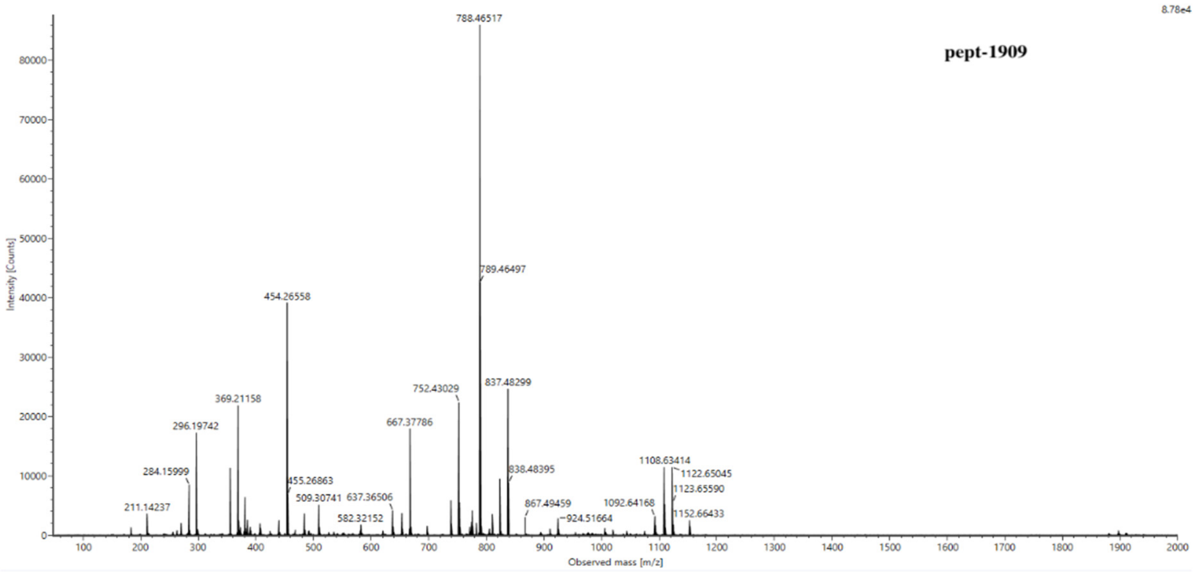
pept-1909
8.78e4
Intensity [Counts]
Observed mass [m/z]
788.46517
789.46497
454.26558
455.26863
837.48299
838.48395
752.43029
667.37786
637.36506
582.32152
509.30741
369.21158
296.19742
284.15999
211.14237
867.49459
924.51664
1092.64168
1108.63414
1122.65045
1123.65590
1152.66433

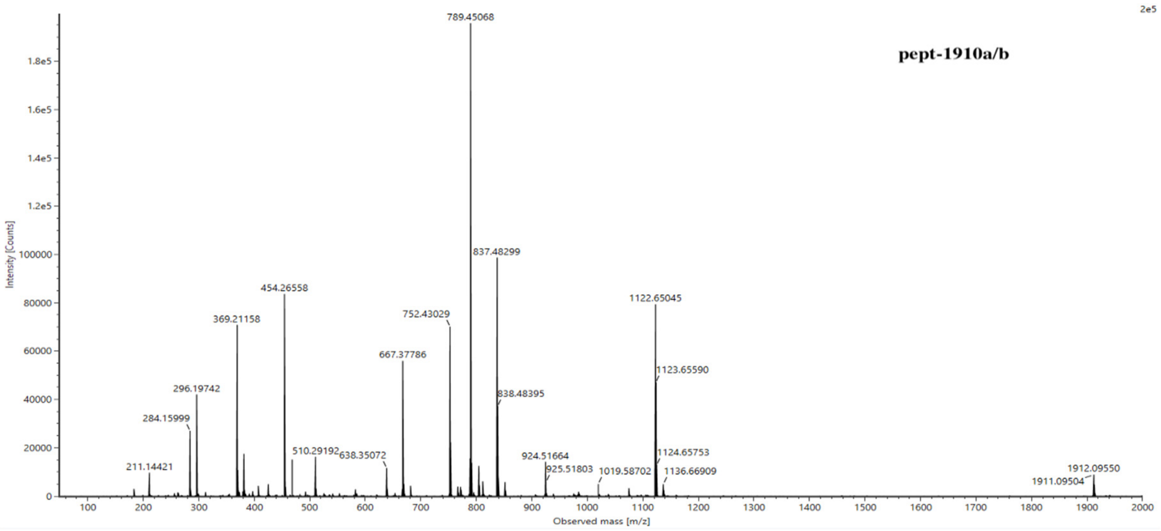
pept-1910a/b
2e5
Intensity [Counts]
Observed mass [m/z]
789.45068
837.48299
838.48395
454.26558
369.21158
752.43029
667.37786
296.19742
284.15999
211.14421
510.29192
638.35072
924.51664
925.51803
1019.58702
1122.65045
1123.65590
1124.65753
1136.66909
1912.09550
1911.09504

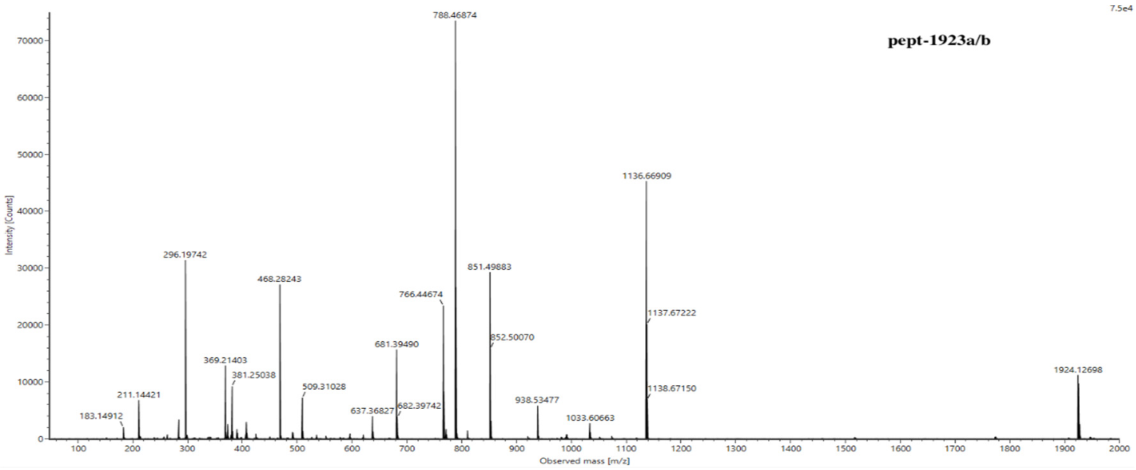
pept-1923a/b
7.5e4
788.46874
1136.66909
296.19742
468.28243
851.49883
766.44674
1137.67222
852.50070
369.21403
381.25038
681.39490
211.14421
509.31028
1924.12698
183.14912
637.36827
682.39742
938.53477
1033.60663
1138.67150
Intensity [Counts]
Observed mass [m/z]

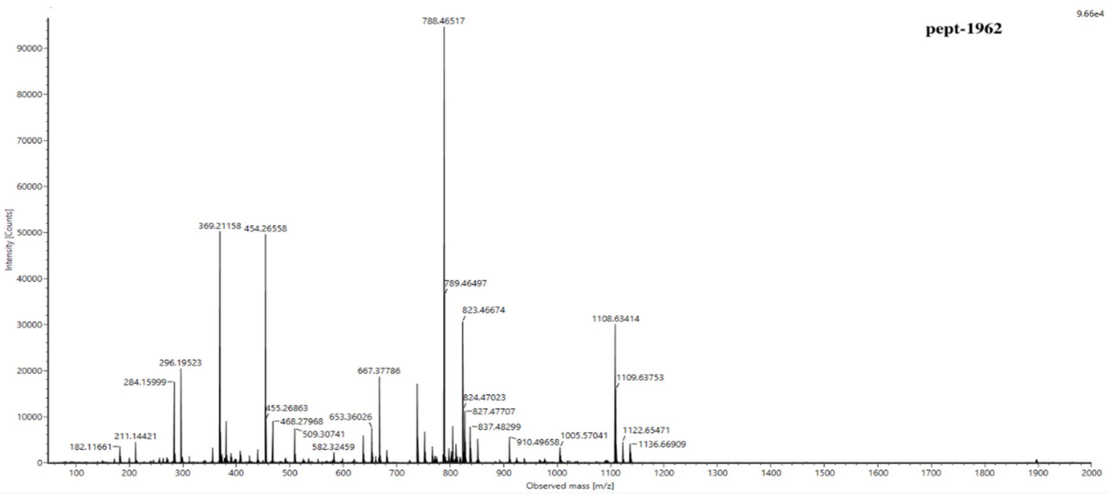
pept-1962
9.66e4
788.46517
369.21158
454.26558
789.46497
823.46674
1108.63414
296.19523
284.15999
667.37786
1109.63753
824.47023
455.26863
827.47707
468.27968
653.36026
837.48299
509.30741
1122.65471
211.14421
1005.57041
182.11661
582.32459
910.49658
1136.66909
Intensity [Counts]
Observed mass [m/z]
